# Fractional CO_2_ Laser for Pediatric Hypertrophic Scars: Lessons Learned from a Prematurely Terminated Split-Scar Trial

**DOI:** 10.3390/ebj6010010

**Published:** 2025-02-20

**Authors:** Sarthak Sinha, Altay Baykan, Karen Hulin, Doug Baron, Vincent Gabriel, Frankie O. G. Fraulin

**Affiliations:** 1 Cumming School of Medicine, University of Calgary, Calgary, AB T2N 1N4, Canada; sarthak.chinoo@gmail.com; 2 Section of Pediatric Surgery, Department of Surgery, University of Calgary, Calgary, AB T2N 1N4, Canada; baykan1@ualberta.ca; 3 Section of Plastic Surgery, Department of Surgery, University of Calgary, Calgary, AB T2N 1N4, Canada; 4 Department of Rehabilitation, Alberta Children’s Hospital, Calgary, AB T3B 6A8, Canada; karen.hulin@albertahealthservices.ca (K.H.); doug.baron@albertahealthservices.ca (D.B.); 5 Department of Clinical Neurosciences, University of Calgary, Calgary, AB T2N 1N4, Canada

**Keywords:** pediatric scars, hypertrophic scars, CO_2_ laser, split-scar design, scar maturity, cutometer, scar biomechanics, Vancouver Scar Scale

## Abstract

Background: Assessing hypertrophic scar (HTS) interventions is challenging because scars continue to undergo dynamic changes. A split-scar design can distinguish treatment effects from natural HTS evolution. Despite promising reports of ablative fractional CO_2_ lasers (AFCO_2_Ls) for HTS, split-scar evidence, particularly in pediatric scars, remains limited. Objective: To explore the feasibility of a split-scar design in assessing AFCO_2_L’s impact on pediatric HTS and to identify potential trends in treatment outcomes. Methods: Initially designed as a prospective single-center split-scar randomized controlled trial, our study transitioned to a feasibility trial due to recruitment challenges. Pediatric patients aged 1–17 years with HTS suitable for split-scar evaluation received three AFCO_2_L treatments at 6–8-week intervals, with outcomes assessed using the Vancouver Scar Scale (VSS), SCAR-Q, and Cutometer. Results: Recruitment was limited by COVID-19 restrictions, concerns about general anesthesia for split-scar treatment, and low interest in divided-scar interventions, resulting in only 6 participants with 9 scars enrolled, far below the target sample size of 44. This small heterogeneous sample precluded meaningful clinical outcome analysis. Conclusions: Our feasibility trial highlights challenges in conducting rigorous pediatric HTS studies and the need for careful interpretation of evidence due to potential publication bias. Future trials should focus on tailored recruitment and comprehensive reporting to improve feasibility and reliability.

## 1. Introduction

Hypertrophic scarring (HTS) is a fibroproliferative condition resulting from deep dermal injuries such as burns, surgery, or trauma. These injuries trigger an intensified inflammatory phase of wound healing, in which fibroblasts overproduce extracellular matrix and proliferation signals [1]. Consequently, HTS manifests as raised, painful, pruritic, and contractile scar tissue that restricts movement, imposes growth restrictions in children, and inflicts enduring functional, aesthetic, and psychosocial issues [2,3]. Adolescents are especially prone to HTS [4,5], possibly due to prolonged wound remodeling. Despite the USD 16 billion annual market for anti-scarring therapies [6], current treatments fall short, especially for children who face heightened risk of scarring and associated symptoms.

Fractional lasers offer a promising approach to HTS treatment by creating a pattern of microscopic thermal injuries that leaves some untreated areas to drive repair [7]. Fractional lasers can be non-ablative (e.g., Erbium (Er) glass laser, 1550 nm wavelength) or ablative (e.g., CO_2_ laser, 10,600 nm wavelength, Er: yttrium aluminum garnet (YAG) laser, 2940 nm wavelength). Non-ablative lasers produce columns of coagulated tissue comprising denatured ECM while preserving the epithelium. Conversely, ablative lasers create columns of vaporized tissue surrounded by eschar and coagulated tissue. This triggers a sterile repair response involving the suppression of matrix synthesis with concomitant activation of proteases like matrix metalloproteinase I [8], which promotes HTS regression. Treatment algorithms have begun recommending AFCO_2_L, starting 9 months post-burn for adults and children [9] and with vascular lasers for mature HTS [10]. Early intervention using AFCO_2_L within the first three months of burn injuries can prevent pathological scarring [11]. However, current evidence is largely retrospective. A meta-analysis reviewing pediatric HTS treatment with AFCO_2_L found that of 10 studies recruiting 413 children [12], only 2 [13,14] were randomized controlled trials (RCTs), with only 1 split-scar design [13]. While AFCO_2_L shows promise, this meta-analysis did not assess publication bias, potentially masking negative outcomes.

Considering that HTS develops over time [15] and mostly regresses without intervention [16], a split-scar design is the gold standard for testing scar interventions as it enables discernment of treatment effects from natural scar progression [17]. However, split-scar evidence, especially for pediatric scars across varying ages, scar maturity, and laser settings, remains scarce. This study explored the feasibility of an evaluator-blinded split-scar trial assessing AFCO_2_L in pediatric HTS. Additionally, we re-examined the existing AFCO_2_L literature for signs of selective publication, a potential bias that could overstate the benefits of laser therapy.

## 2. Materials and Methods

### 2.1. Study Design

Originally designed as a single-center split-scar randomized controlled trial, the study transitioned to a feasibility trial due to recruitment challenges, including COVID-19 restrictions and concerns about general anesthesia for split-scar treatments. The revised objective focused on assessing the practicality of using a split-scar design to evaluate the effects of AFCO_2_L on pediatric HTS. Approved by the Conjoint Health Research Ethics Board (REB17-2362) on 2 March 2020 and registered on ClinicalTrials.gov (NCT04236167), the study was prematurely terminated in November 2022 after enrolling six patients.

### 2.2. Participants

Six female patients (ages 5–17.3 years, scar ages 0.5–15.4 years) were enrolled. Their skin types spanned Fitzpatrick classifications II to VI, and scar causes included four scalds and two contact injuries (Table 1). The inclusion criteria were patients aged 1 to 17 years, diagnosed with hypertrophic scars from acute injuries or burns, and stable closed scars for at least three months post-wound closure. The exclusion criteria were contraindications to general anesthesia, open wounds, active infections, prior CO_2_ laser therapy on scar, or skin disorders impacting wound healing.

### 2.3. Laser Treatments and Study Protocol

Each scar was divided into two sections for a controlled comparison. Scar sections were matched for size, appearance, VSS, and Cutometer measurements. These halves were assigned unique “Site IDs” on a transparency map to enable longitudinal tracking. Treatment allocation to each half was randomized across patients to ensure unbiased assessments by blinded evaluators A.B. and K.H. While K.H. was involved in clinical care, A.B. was not. To maximize inter-rater reliability, A.B. and K.H. calibrated a series of scar measurements over time, practicing on non-study patients before the trial commenced. Orthogonal measurements were taken when feasible (e.g., with the Cutometer) to enhance reproducibility and accuracy. All laser procedures were carried out at the Alberta Children’s Hospital in the main operating room under general anesthesia. Senior author (F.O.G.F.) performed treatments using the UltraPulse CO_2_ Laser (Lumenis, Yokneam, Israel) in three sessions 6–8 weeks apart. The treatment parameters were SCAAR FX mode targeting up to a depth of 4 mm with settings ranging from 70–150 mJ in energy, 1–5% in density, and 150–250 Hz in frequency. Following this, the Deep FX mode treated superficial scar layers up to 1 mm depth (12.5–22.5 mJ, 5–15%, 300–600 Hz). The active FX mode was reserved for patients with surface-level irregularities, using a single pass (80–125 mJ, 2–3%, 100–150 Hz). Post-op care included acetaminophen for pain and petroleum jelly for wound care. Patients were instructed to report fever, pain escalation, or wound issues.

### 2.4. Outcome Measures

We recorded the characteristics of our study sample including patient age, Fitzpatrick skin type, burn date, mechanism, location, TBSA, and prior skin grafting. For evaluating scar progression, we used the validated Vancouver Scar Scale (VSS) [18] at three time points: before the initial treatment, immediately before the second treatment, and 4–8 weeks after the final session. Along with standard VSS parameters, which include vascularity, pigmentation, pliability, and height, we incorporated a 0–2 scale to assess pain and itch, where 0 indicates no symptoms, 1 indicates occasional symptoms, and 2 indicates symptoms requiring medication, as seen in other studies [19]. For the same timepoints, we utilized the MPA 580 Cutometer (Courage + Khazaka electronic GmbH, Köln, Germany) to non-invasively quantify the viscoelastic properties of scars. The device’s software, Cutometer Dual version 2.2.2.1, captured 10 measurements (R0–R9) indicating the different mechanical properties of the scar. We recorded and analyzed R0 (firmness) and R1 and R2 (elasticity). Employing consistent settings, the Cutometer applied a 450 mbar suction to small skin areas and measured deformation over a 4-s cycle with 3 repetitions—2 s of suction followed by 2 s of release. Measurements were taken in 3 adjacent sites for both control and laser-treated scars, following established methods for in vivo measurements [20]. Each site was marked on a transparency map to enhance the reliability of longitudinal assessment [21,22].

### 2.5. Data Analysis

Data analysis was performed by S.S. and A.B., neither of whom were involved in patient care.

Due to the trial’s exploratory nature and small sample, analyses were descriptive. Rather than applying formal hypothesis tests or reporting *p*-values, our intent was to observe possible trends in outcomes across the small and heterogeneous sample. Numerical changes in scar characteristics, Cutometer measures, and patient-reported outcomes were summarized, and comparisons were presented narratively.

To explore potential trends in treatment response, we employed KMeans clustering as an exploratory tool based on baseline VSS scores and scar age. These analyses aimed to identify patterns that could inform future research rather than draw definitive conclusions. To discern scar subgroups that may show a discrepant laser response, we utilized the KMeans algorithm from scikit-learn v.1.4.1 (sklearn.cluster), setting n_clusters to 4 and initializing with a random state of 0. We normalized each scar’s data before clustering to optimize performance. Clusters were added as a ‘Cluster’ column in the dataset. We evaluated the laser treatment effectiveness across these clusters by comparing average changes in VSS and Cutometer measurements from the baseline to final follow-up between treated versus control scars across clusters. For ‘responder’ versus ‘non-responder’ analysis, we classified scars as responders if they showed improvement exceeding one standard deviation from mean across more than half the variables measured, achieving a conservative yet clinically meaningful distinction. This method identified 44.44% of control and 22.22% of the laser scars as ‘responders’. To identify predictors of the ‘responder’ group, we conducted univariate and multivariate analyses, examining variables such as scar age, burn etiology, anatomical location of the scar, and baseline measurements of pigmentation, vascularity, pliability, and height.

### 2.6. Publication Bias Assessment

To contextualize our findings, we assessed publication bias in the existing AFCO_2_L literature for pediatric HTS. We re-analyzed 5 studies pooled in Chen et al. 2024 [12] for VSS outcomes with regard to pliability, pigmentation, vascularity, and height. We extracted effect sizes, confidence intervals (CIs), and sample sizes (Supplemental Table S1). We used Egger’s regression to detect publication bias by examining the asymmetry of funnel plots, which depicts the relationship between study effect sizes and precision (inverse of the standard error (SE)). SE was calculated from 95% CIs. Funnel plots were generated using the *seaborn* library and Egger’s regression was performed using *scipy* library’s *stats.linregress* function. A *p*-value under 0.05 was considered significant for publication bias.

## 3. Results

### 3.1. Patient Demographics

A-priori power analysis projected 44 pediatric HTS patients for detecting meaningful improvement. We aimed to recruit patients over 2 years from our outpatient burn and plastic surgery clinics at Alberta Children’s Hospital. Despite these targets, from October 2020 to November 2022, we encountered significant recruitment challenges due to COVID-19 restrictions, limited enrolment for split-scar treatment, and a lower-than-anticipated number of eligible patients. Consequently, our study’s sample size fell short of what was required for powered post-hoc analyses, recruiting only six pediatric female participants with nine HTS. Participants were 5.0–17.3 years old (median 7.5); scars ranged from 0.5–15.4 years (median 5.3). Skin types spanned Fitzpatrick Types II through VI, with most scars located on upper extremities, and the burn mechanisms included four scalds and two contact burns (Table 1). Given the small and heterogeneous sample, the study’s findings should be interpreted as exploratory.

### 3.2. Assessment of the AFCO_2_L Effect Using VSS

We compared baseline characteristics between control and laser-treated split-scars across VSS domains. Both groups demonstrated comparable baseline characteristics, with mean scores of 1.67 for pigmentation, 0.67 for vascularity, 2.56 (control) and 2.67 (laser) for pliability, and 1.22 (control) and 1.11 (laser) for height. The mean scores for pain and itch were 0.33 and 0.56, respectively. No formal statistical testing was performed due to the small sample size and feasibility design. Visual inspection confirmed similar baseline characteristics.

After the first treatment, control scars had mean scores of 1.44 for pigmentation, 0.67 for vascularity, 2.33 for pliability, and 1.33 for height, with pain and itch registering minimal scores indicating minimal discomfort or irritation post-treatment. Laser-treated scars had mean scores of 1.56 for pigmentation, 0.89 for vascularity, 2.44 for pliability, and 1.11 for height, with negligible scores for pain and itch. Overall, both groups showed only slight changes from the baseline. These observations suggest that immediately following treatment, the control and laser-treated scars were largely comparable.

After three treatments, the control group exhibited mean scores of 1.33 for pigmentation, 0.33 for vascularity, 1.67 for pliability, and 1.11 for height, with pain and itch presenting minimal scores of 0.11 and 0.67, respectively. Conversely, laser-treated scars displayed elevated mean scores: 1.78 for pigmentation, 1.22 for vascularity, 2.22 for pliability, and 1.33 for height, while reporting low scores for pain and itch, identical to the control scar group. Laser-treated scars appeared to improve less in certain areas, such as vascularity, but overall differences between groups were minimal. Given this study’s small feasibility-focused design, any observed trends should be interpreted with caution. Both groups reported negligible pain and itch.

Finally, at six months post-treatment, the control group’s scars showed mean scores of 0.78 for pigmentation, 0.44 for vascularity, 1.44 for pliability, and 0.78 for height, with no reported pain and a minimal itch score of 0.33. In comparison, laser-treated scars exhibited mean scores of 1.22 for pigmentation, 0.56 for vascularity, 1.78 for pliability, and 1.00 for height, with no pain and a slightly higher itch score of 0.44. Overall, these results indicate that both groups remained broadly comparable, with minimal pain and itch (Table 2).

### 3.3. Assessment of the AFCO_2_L Effect Using a Cutometer

At baseline, control and laser-treated split-scars exhibited similar mean values for R0 (0.130 and 0.132, respectively), R1 (0.028 and 0.031, respectively), and R2 (0.758 and 0.770, respectively), suggesting a uniform starting point. Healthy skin demonstrated higher values (R0 mean of 0.280, R1 mean of 0.033, and R2 mean of 0.869), consistent with expectations of non-scarred skin. Although no formal statistical testing was performed, these observations suggest a uniform starting point between the two groups.

Over the course of treatment, laser-treated scars showed a minor decrease in mean R0 (measurement of pliability), dropping by approximately 0.021 from baseline to the end of the third treatment. Control scars exhibited a slightly larger decrease of about 0.032 (Table 3). For R1, reflecting the skin’s ability to return to its original state, both groups recorded small reductions, with laser-treated scars decreasing by 0.018 and controls by 0.010. Both groups showed slight increases in R2, which measures gross elasticity, with laser-treated scars rising by 0.017 and control scars by 0.022.

For the interested reader, see the Supplementary Material for exploratory cluster analyses of scar outcomes related to maturation time. These preliminary observations emphasize the importance of considering scar age and maturity.

### 3.4. Assessment of the Cluster-Specific Effects of AFCO_2_L Using Scar-Q

SCAR-Q is a validated patient-reported outcome measure designed to evaluate scars from the patient’s perspective [23]. It assesses three main domains: Appearance, Symptoms, and Psychosocial Impact, with a lower score being better. For participants 8 and older, the SCAR-Q was self-completed; for those under 8, it was completed by a parent or guardian, with the same individual completing both pre- and post-treatment assessments for consistency.

**Figure 1:**
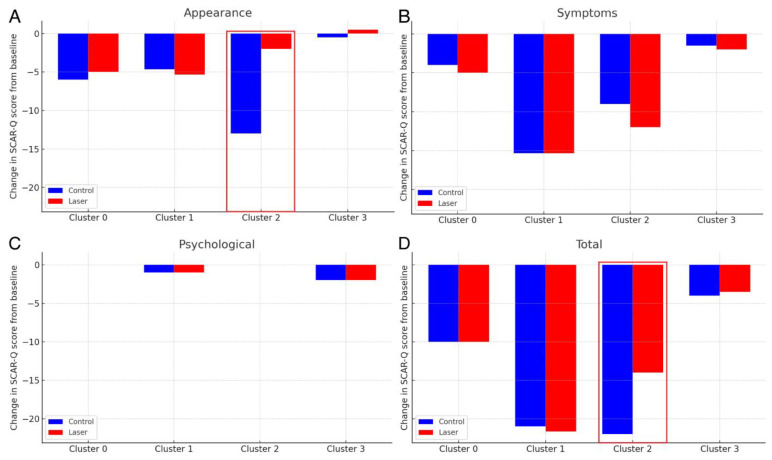
Changes in SCAR-Q scores by cluster and treatment type. Changes in SCAR-Q appearance (**A**), symptoms (**B**), psychological (**C**), and total (**D**) scores from baseline to six months post-treatment.

Our analysis of SCAR-Q scores revealed patterns generally consistent with the VSS and Cutometer results. Younger scar clusters (Clusters 1 and 2) showed more notable changes in total SCAR-Q scores, with both control and laser-treated groups in Cluster 1 decreasing by about 21 points, and Cluster 2 exhibiting a 22-point reduction in the control group versus a 14-point reduction in the laser group (Figure 1). In contrast, older scar clusters (Clusters 0 and 3) displayed smaller shifts, around 10 points in Cluster 0 and roughly 4 points in Cluster 3 for both control and laser-treated scars.

Specifically, within Cluster 2, control scars improved more substantially, decreasing from a total SCAR-Q score of 101 to 79 over six months, while laser-treated scars improved to a lesser extent, from 101 to 87. This more modest improvement in the laser-treated scars suggests that AFCO_2_L may have disrupted the natural course of scar healing, particularly in the appearance domain. While intriguing, these findings require cautious interpretation due to the small sample and exploratory design. While laser treatment did not overtly worsen SCAR-Q outcomes in younger scars, it may have limited the degree of natural improvement seen in the untreated control segments.

### 3.5. Publication Bias in Studies Reporting AFCO_2_L for Pediatric HTS

**Figure 2:**
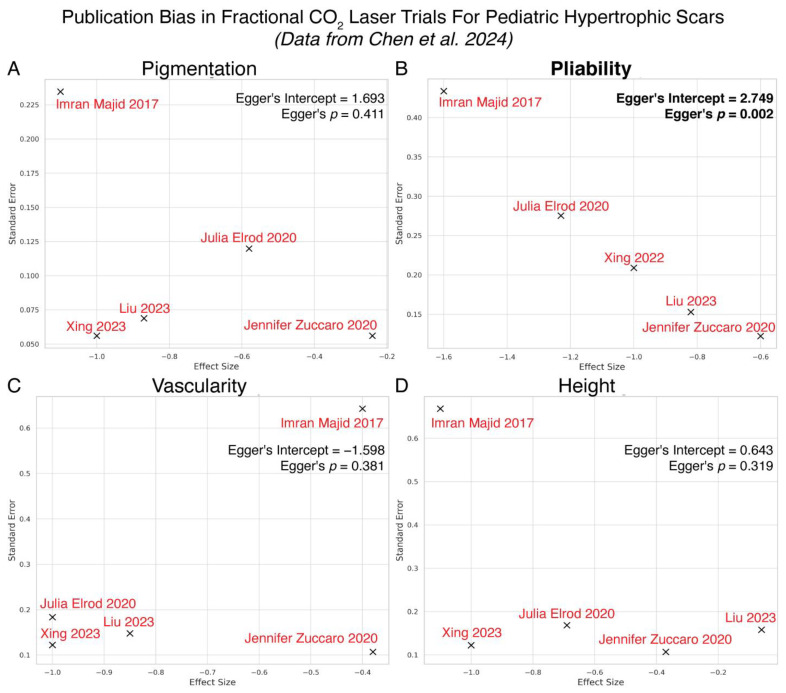
Re-assessment of meta-analysis on AFCO_2_L’s impact on pediatric HTS reveals publication bias for scar pliability. These studies, cited as Majid et al. [24], Elrod et al. [25], Jisong et al. [14], Fuxi et al. [26], and Zuccaro et al. [27], explored effects on scar pigmentation (**A**), pliability (**B**), vascularity (**C**), and height (**D**). Data were extracted from meta-analysis by Chen et al. [12].

Given the paucity of published studies reporting neutral or net negative effects of AFCO_2_L on pediatric HTS, we re-analyzed Chen et al.’s 2024 meta-analysis [12] for publication bias. Indeed, Egger’s regression revealed statistically significant publication bias across studies assessing pliability (Egger’s intercept = 2.7491, *p* = 0.0023) suggesting a discrepancy in the literature where studies reporting negligible or detrimental AFCO_2_L effects are systematically underrepresented. The remaining VSS domains, pigmentation (intercept = 1.6932, *p* = 0.4113), vascularity (intercept = −1.5984, *p* = 0.3808), and height (intercept = 0.6432, *p* = 0.3185), showed no evidence of bias (Figure 2).

## 4. Discussion

Our study, originally designed as a randomized controlled trial, encountered significant recruitment challenges that led to premature termination. Presented as a feasibility trial, these findings provide valuable insights for future studies.

A major factor contributing to the premature termination of our trial was insufficient patient recruitment, a common hurdle in clinical trials across plastic surgery [28] and burn [29] research. Prior surveys highlight recruitment issues as the leading cause of premature trial termination, yet specific barriers to enrollment remain underexplored. We encountered some universal challenges, such as recruitment delays due to the COVID-19 pandemic [30] and others inherent to our study design. For instance, parents and patients expressed a preference for whole-scar treatment, particularly since general anesthesia is typically required for pediatric laser treatments [25,31]. Conversely, adult laser treatments are often performed in an office with topical anesthetics [32]. Similar hesitancy toward split-scar designs has been reported in other unpublished trials (e.g., NCT02776618, NCT01995604), where recruitment issues ultimately led to early termination. By documenting these challenges, we provide insights into the feasibility of split-scar studies and highlight the importance of patient and parent engagement in trial planning.

Our findings also highlight the need to refine patient selection algorithms to optimize trial power, acknowledging the ubiquity of patient recruitment challenges in research. Based on preliminary cluster analysis results, we suggest prioritizing older scars with significant pliability issues for AFCO_2_L intervention. These scars, typically over 10 years old, may benefit from the laser’s remodeling effects, particularly when pliability is the chief concern. Conversely, younger scars, especially those less than five years old, may require a more conservative approach. For these patients, other interventions or observation may be more appropriate until the scar reaches a more mature stage. Our preliminary observations of a heterogeneous treatment effect are corroborated by our reappraisal of a recent meta-analysis [12], where authors noted significant overall treatment effects, but our re-analysis suggested an underappreciated publication bias that obscures negative findings. This bias is exemplified by terminated trials such as NCT01826942 and NCT01654406, which were halted due to issues including worsening of scar, and by studies like NCT00969215 that were completed but remain unpublished [29].

Variability in AFCO_2_L parameters across studies must also be considered, as energy density, pulse width, and spot diameter critically influence the thermal effects on HTS [33]. In their split-scar RCT, Won et al. employed a low-energy AFCO_2_L setting (30 mJ/pixel) on children averaging two years of age [13], reporting positive outcomes. This study contrasts with our approach, utilizing the UltraPulse^®^ Lumenis AFCO_2_L with SCAAR FX™ designed specifically for deep scar penetration [34] at higher energy levels (70–150 mJ). Our settings are more similar to those used in adults (70–125 mJ) [35,36]. While future trials can consider addressing recruitment challenges by adopting a multi-center approach, standardization poses significant logistical challenges. The cost of AFCO_2_L is approximately USD 225,000 CAD, with yearly maintenance costs of roughly USD 5000 CAD. Collaboration would require centralized equipment purchasing equipment, standardized training, and protocol alignment. Future trials should also prioritize objective assessment tools, combining the Cutometer with 3D photogrammetry [37], ultrasonography [38,39,40], and scar-biopsy histology [41] to enable more comprehensive analysis.

Generalizability is limited by the small single-center sample of six female participants. There may be some bias from parental completion of the SCAR-Q for participants under 8 years old. While validated for self-reporting in children 8 and older, younger participants required parental assistance, which may have introduced discrepancies, particularly in psychosocial domains. Finally, it is possible that treating half of the scar may have inadvertently induced broader cutaneous changes that affect the untreated portion.

While our feasibility study did not yield definitive conclusions on the efficacy of AFCO_2_L in treating pediatric hypertrophic scars, it underscores the importance of scar maturity and individualized treatment protocols. Varied responses to laser therapy observed in our study suggest that further research is necessary to optimize patient selection and treatment parameters. Future studies should focus on establishing clear guidelines for AFCO_2_L in different scar types and ages, with the goal of improving outcomes for children with hypertrophic scars.

## 5. Conclusions

While split-scar assessment remains the gold standard for evaluating pediatric hypertrophic scar intervention due to their ability to account for natural scar evolution, our study highlights the significant challenges in conducting such trials, including recruitment difficulties and hesitancy toward split-scar treatments. Overcoming these obstacles will require multicenter collaborations to increase sample sizes with standardized protocols for laser treatments and scar assessments.

## Figures and Tables

**Table 1:** Characteristics of split-scar study participants. Abbreviations: TBSA: Total Body Surface Area; y: years; L: left; R: right.

Patient	Age (y)	Scar Age (y)	Location	TBSA	Grafted	TBSA Grafted	Mechanism	Skin Type
1	5	1.1	Back, L shoulder	14%	Yes	5%	Scald	VI
2	14.3	13.5	L/R palms	2%	Yes	2%	Contact	II
3	17.3	15.4	R arm	4%	No	0%	Contact	V
4	5.8	3.9	R arm	14%	Yes	4%	Scald	III
5	8.7	6.7	L arm	4%	No	0%	Scald	IV
6	6.4	0.5	Pubic area	2%	No	0%	Scald	V

**Table 2:** Summary of Vancouver Scar Scale (VSS) scores across 9 split-scar sites post 1st and 3rd treatments. Values presented represent mean and standard deviation (SD) of VSS scores for control and laser-treated scars.

	Baseline	After 1st Treatment	After 3rd Treatment	Change (After 1st Treatment)	Change (After All Treatment)
Control	7.00 (2.06)	6.11 (1.62)	5.22 (2.54)	−0.89	−1.78
Laser	7.00 (2.18)	6.33 (1.80)	7.33 (2.29)	−0.67	+0.33

**Table 3:** Summary of cutometer measurements across nine split-scar sites. Abbreviations: Total Extensibility (R0), Elastic Recovery (R1), and Elasticity Ratio (R2 = R1/R0).

	Baseline	After 1st Treatment	After 3rd Treatment	Change (After 1st Treatment)	Change (After All Treatment)
R0 (Control)	0.130	0.112	0.098	−0.018	−0.032
R0 (Laser)	0.132	0.148	0.111	+0.018	−0.021
R1 (Control)	0.028	0.018	0.018	−0.010	−0.010
R1 (Laser)	0.031	0.032	0.013	+0.001	−0.018
R2 (Control)	0.758	0.833	0.790	+0.075	+0.022
R2 (Laser)	0.770	0.789	0.787	+0.019	+0.017

## Data Availability

The original contributions presented in the study are included in the article/Supplementary Material; further inquiries can be directed to the corresponding authors.

## Supplementary Materials

The following supporting information can be downloaded at https://www.mdpi.com/article/10.3390/ebj6010010/s1: Figure S1: Cluster analysis of laser-treated pediatric scars illustrates potential trends in response based on scar maturity, serving as an exploratory example of analyses feasible in larger well-powered trials; Figure S2: Schematic highlighting hypothetical pathways to identify mature scars with pathological pliability that may benefit from future laser therapy trials; Supplementary Table S1: Egger’s test results and extracted study data for AFCO_2_L in pediatric HTS. The table summarizes effect sizes, confidence intervals, and sample sizes extracted from Chen et al. 2024 [12] along with Egger’s test results assessing publication bias across four VSS domains.
