# Supplementary material for: Fractional CO2 Laser for Pediatric Hypertrophic Scars: Lessons Learned from a Prematurely Terminated Split-Scar Trial
Source: Eur Burn J. 2025 Feb 20;6(1):10. doi: 10.3390/ebj6010010 (PMC11941394; doi:10.3390/ebj6010010)
Supplement: Supplementary file 1 [file ebj-06-00010-s001.zip › Supplementary.docx]

**Re-assessment of AFCO_2_L efficacy across scar clusters**

To explore potential trends in treatment response, scars were grouped into four clusters based on baseline VSS scores and scar age. Cluster 0 (4 scars) had hypopigmentation, normal vascularity, high pliability, slight height, and no pain or itch, with a mean scar age averaging 13.5 years. Cluster 1 (6 scars) showed mixed pigmentation, pink vascularity, moderate pliability, minimal height, and some pain and itch, averaging around 2.0 years post-injury. Cluster 2 (4 scars) also had mixed pigmentation and pink vascularity but presented firmer pliability and moderate height, with no pain but some itch, and represented the youngest group at about 0.8 years old. Cluster 3 (4 scars) displayed mixed pigmentation, slightly pink vascularity, yielding pliability, minimal height, and no pain or itch, with a mean scar age around 11.1 years (**Figure S1A**).

In Cluster 0, AFCO_2_L treatment appeared to enhance pliability more than control (approximately a 2.5-point improvement vs. 1-point in controls). In contrast, Cluster 2 demonstrated a scenario where the control scars naturally improved across multiple VSS parameters (e.g., pliability decreased by 1 point, pigmentation by 2 points, vascularity by 0.5, height by 1), while laser-treated scars showed little to no corresponding improvement and reduced gains in pigmentation (**Figure 1B**). The Cutometer findings for Cluster 2 supported this pattern, with control scars showing an increase in elasticity while AFCO_2_L-treated scars did not, suggesting that AFCO_2_L may have disrupted the natural healing trajectory in these younger scars (**Figure S1C-D**).

These preliminary observations emphasize the importance of considering scar age and maturity. For instance, older scars with initially high pliability (as seen in Cluster 0) seemed to benefit more from AFCO_2_L, whereas younger scars (like those in Cluster 2) may experience disrupted remodeling with this treatment. When assessing factors such as scar age, etiology, location, and baseline characteristics, scar age emerged as an especially relevant factor in predicting favorable responses to AFCO_2_L (**Figure S1E**). Future studies may focus on patients with older scars, where the primary issue is reduced pliability, to potentially optimize the therapeutic effects of AFCO_2_L. However, further research is necessary to confirm these findings, refine patient selection criteria, and determine the best timing for intervention (**Figure S2**).
