# Supplementary figures and images for: Fractional CO2 Laser for Pediatric Hypertrophic Scars: Lessons Learned from a Prematurely Terminated Split-Scar Trial
Source: Eur Burn J. 2025 Feb 20;6(1):10. doi: 10.3390/ebj6010010 (PMC11941394; doi:10.3390/ebj6010010)

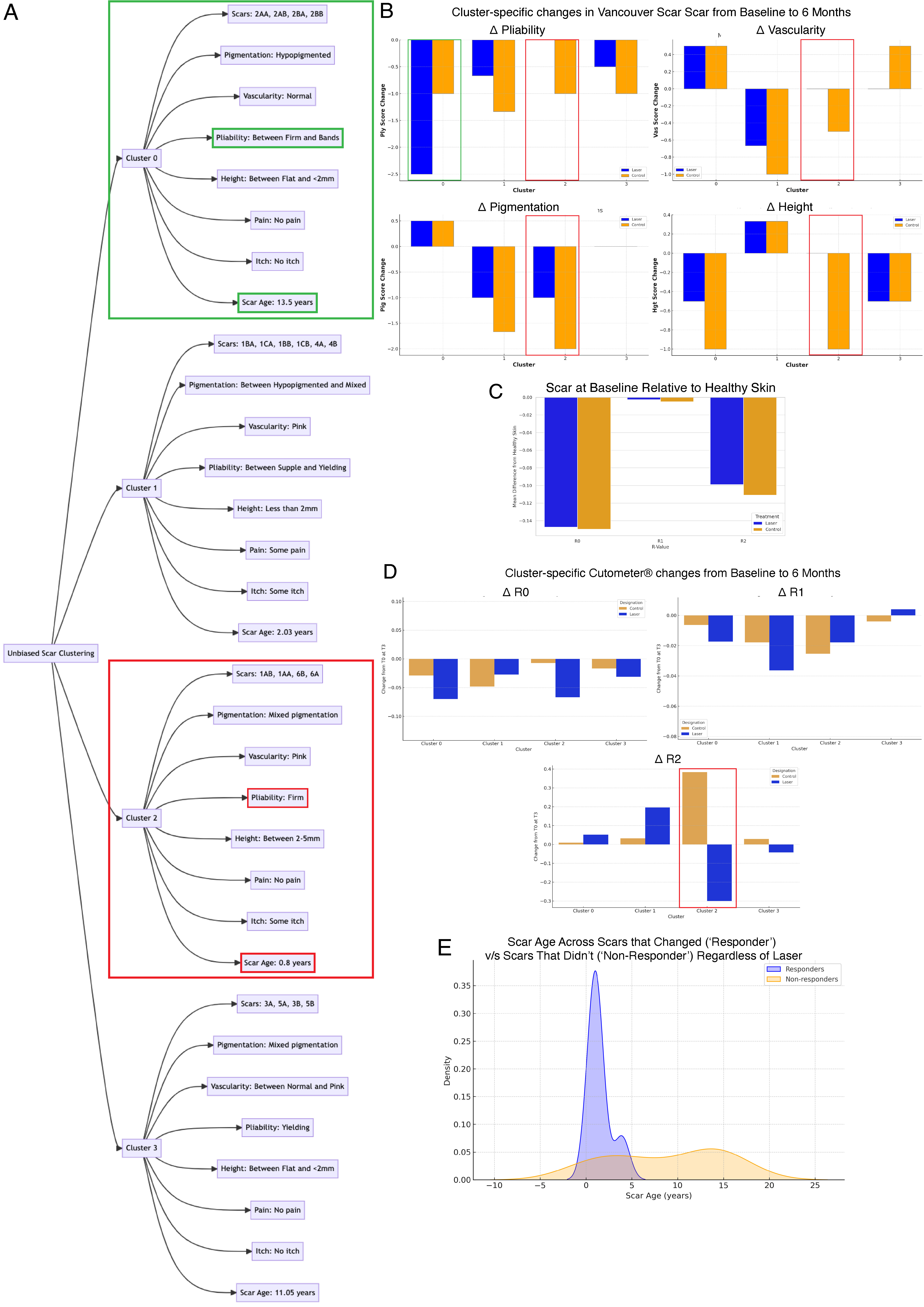

Supplement: Supplementary file 1 [file ebj-06-00010-s001.zip › SFig_1_Pediatric_Laser_Scar_Clustering.jpg]

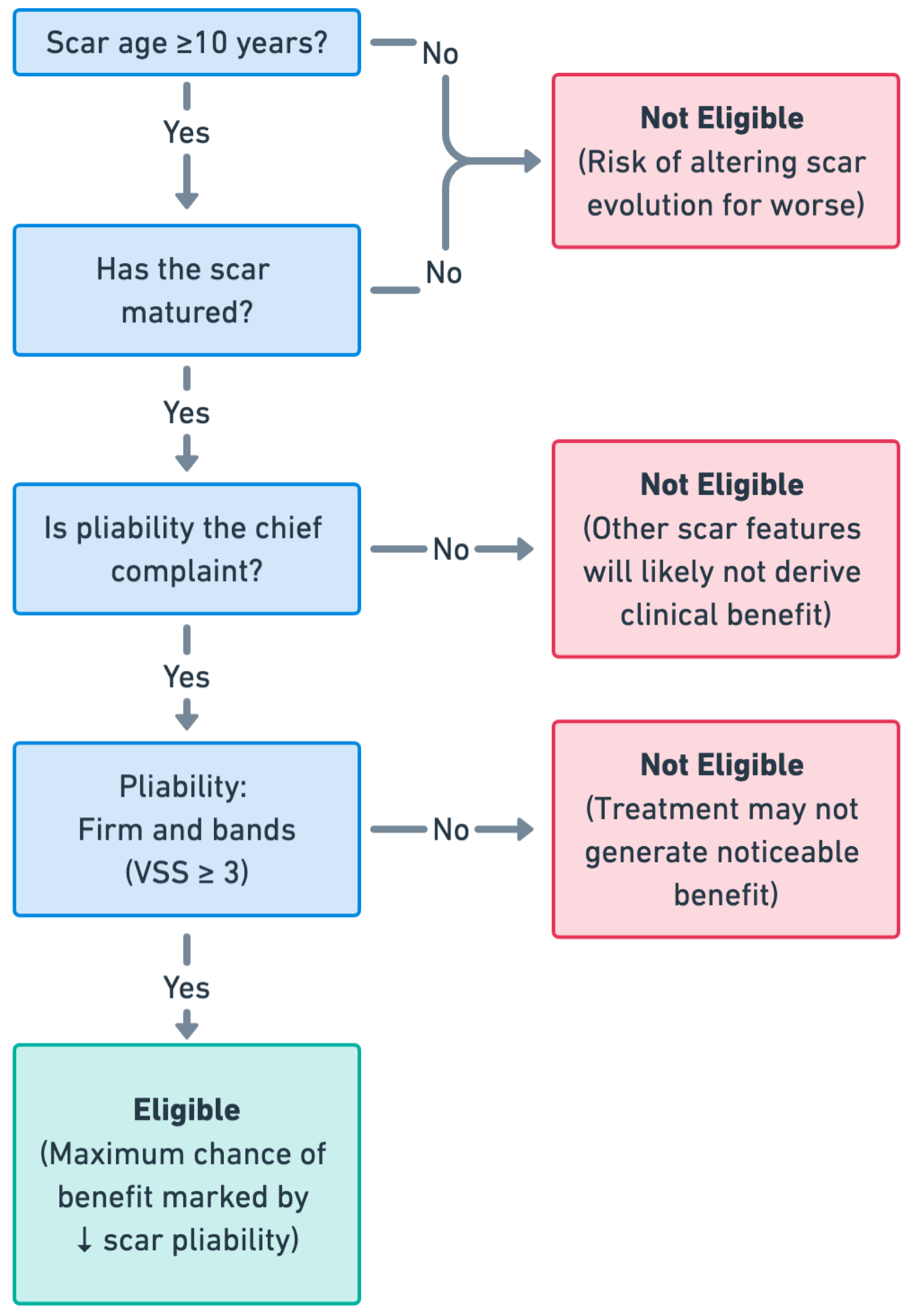

Supplement: Supplementary file 1 [file ebj-06-00010-s001.zip › SFig_2_Recruitment_Approch.jpg]
